# Non-Viral Episomal Vector Mediates Efficient Gene Transfer of the β-Globin Gene into K562 and Human Haematopoietic Progenitor Cells

**DOI:** 10.3390/genes14091774

**Published:** 2023-09-08

**Authors:** Vassileios M. Lazaris, Emmanouil Simantirakis, Eleana F. Stavrou, Meletios Verras, Argyro Sgourou, Maria K. Keramida, George Vassilopoulos, Aglaia Athanassiadou

**Affiliations:** 1 Department of General Biology, Medical School, University of Patras, 26504 Patras, Greece; vlazaris@gmail.com (V.M.L.); stauroue@upatras.gr (E.F.S.); meletver@gmail.com (M.V.); 2 Centre of Basic Research, Biomedical Research Foundation of the Academy of Athens (BRFAA), 11527 Athens, Greece; esimantirakis@gmail.com (E.S.); gvasilop@bioacademy.gr (G.V.); 3 Biology Laboratory, School of Science and Technology, Hellenic Open University, 26335 Patras, Greece; sgourou@eap.gr; 4 IVF and Andrology Labs, IVF Unit, General University Hospital of Patras, 26504 Patras, Greece; maria_keram@yahoo.gr

**Keywords:** non-viral vector, non-integrating, episomal, β-globin, β-Thalassemia, S/MAR, IR, gene therapy

## Abstract

β-Thalassemia is a subgroup of inherited blood disorders associated with mild to severe anemia with few and limited conventional therapy options. Lately, lentiviral vector-based gene therapy has been successfully applied for disease treatment. However, the current development of non-viral episomal vectors (EV), non-integrating and non-coding for viral proteins, may be helpful in generating valid alternatives to viral vectors. We constructed a non-viral, episomal vector pEPβ-globin for the physiological β-globin gene based on two human chromosomal elements: the scaffold or matrix attachment region (S/MAR), allowing for long nuclear retention and non-integration and the β-globin replication initiation region (IR), allowing for enhancement of replication and establishment. After nucleofections into K562 cells with a transfection efficiency of 24.62 ± 7.7%, the vector induces stable transfection and is detected in long-term cultures as a non-integrating, circular episome expressing the β-globin gene efficiently. Transfections into CD34+ cells demonstrate an average efficiency of 15.57 ± 11.64%. In the colony-forming cell assay, fluorescent colonies are 92.21%, which is comparable to those transfected with vector pEP-IR at 92.68%. Additionally, fluorescent colonies produce β-globin mRNA at a physiologically 3-fold higher level than the corresponding non-transfected cells. Vector pEPβ-globin provides the basis for the development of therapeutic EV for gene therapy of β-thalassemias.

## 1. Introduction

Gene therapy is currently a promising strategy for the treatment of incurable diseases where only supportive therapy can be administered. The European Medicines Agency (EMA) has approved a few medicinal products, including a vector for the gene therapy of β-thalassemia for non-β^0^/β^0^ genotype of the adult β-globin gene. The human β-globin gene, after the switching of the γ- to β-globin gene expression, is expressed in the adult stage of human life, in the red bone marrow, during erythropoiesis and particularly in reticulocytes and erythrocytes [1].

β-Thalassemia is a severe congenital hemolytic anemia caused by decreased or absent β-globin peptides, mainly due to mutations in the β-globin gene locus. There are at least 350 mutations in the β-globin gene worldwide. As a result, β-thalassemia patients present an impressive diversity of phenotypes that range from transfusion-dependent severe anemia to a clinically asymptomatic state [2]. Currently, the management of β-thalassemia patients is based on blood transfusions and life-long iron chelation. This approach aims to avoid the iron loading that jeopardizes the overall life expectancy and has a negative impact on the outcome of bone marrow transplantation, the only curative procedure for those who happen to have a matching donor [3].

Regardless of the genotype, patients with β-thalassemia can benefit from therapeutic gene addition based on the integrating lentiviral vectors (LV) currently used. However, this approach is not without drawbacks, as the integration of these vectors into the host stem cell DNA may be followed by insertional mutagenesis, which has a risk for oncogenic development [4]. LV may also cause the activation or deactivation of signaling by the tumor suppressor p53 gene [5]. While a possible intervention of plasmids in the function of such genes cannot be excluded, no relative data exist yet. An alternative curative route for the β-thalassemia syndromes relies on CRISPR-Cas9-based genome editing technology [6], which focuses on the correction of mutations such as homozygous β^0^39, one of the most severe β-thalassemia phenotypes. Lentiviral vectors have failed so far to efficiently cure β^0^39 carriers since patients continue to receive blood transfusions, but less frequently and with less load than before their enrolment [7].

An alternative, non-invasive gene addition strategy is exogenous gene transfer by non-viral, episomal vectors that do not integrate and do not interfere with the function of the host DNA. 

The development of non-viral episomal vectors (EV) for therapeutic gene transfer has been advancing over the last few decades [8]. Episomal vectors are plasmids between 5 and 10 kb that bear a number of features that can address issues of genome safety and transgene expression efficiency. In terms of design, current EVs are free of any genes that produce viral proteins, minimizing the possibility of eliciting an immune response. Nevertheless, a number of challenges still remain. A major drawback of the original types of episomal vectors was the occasional integration into the chromosomal DNA and the plasmid loss over time in culture. The latter was most prominent in tissues with high proliferating rates, where it could result in complete loss of the episomes by day 30 [9]. 

A major leap forward in the evolution of non-viral episomal vectors was the construction of the prototype, episomal vector pEPI-1 [10], with the inclusion of the chromosomal element scaffold or matrix attachment region (S/MAR), a DNA sequence that normally resides in the 5′-end of the human β-interferon gene. The S/MAR element blocks the integration of the pEPI-1 plasmid into the chromosomal DNA, thus the cell is avoiding insertional mutagenesis [10]. In addition, cells transfected with vector pEPI-1 may acquire mitotic stability, with the plasmid segregating properly to daughter cells during successive mitoses, preventing plasmid loss [11]. 

Further research has revealed important properties of the S/MAR element, which provide an understanding of the non-integration and mitotic stability of the pEPI-1 vector. The S/MAR was found to be an AT-rich DNA element, which is often in a denatured DNA configuration and tethers the plasmid DNA to sub-nuclear structures through binding to nuclear matrix proteins, e.g., SAF-A [12]. Furthermore, plasmid pEPI-1 and its derivatives replicate autonomously as extra-chromosomal elements just once per cell cycle [13]. Plasmid pEPI-1 carries the S/MAR element as part of the transcription cassette of eGFP (e.g., promoter CMV-eGFP-S/MAR-PolyA site). Consequently, this element is transcribed along with the eGFP gene and is often in a denatured DNA configuration [10]. Therefore, non-integration that prevents insertional mutagenesis and mitotic stability that prevents plasmid loss, even though we cannot yet understand the deep mechanism of their operation, are nevertheless crucial properties of the pEPI-1 plasmid as a non-viral, episomal vector for gene therapy.

A number of derivative vectors from pEPI-1 were constructed and were used to test the S/MAR technology in different settings: (i) episomal expression in mouse liver [14], (ii) elimination of harmful sequences which increases safety, such as in the minicircle [15] in vitro and in vivo [16], in a pre-clinical model [17], in the pEPIto vector [18] and in an in vivo study [19], (iii) efficient episomal gene transfer suitable for liver-directed studies with the pFAR-S/MAR vector [20], (iv) genetic modification of pluripotent stem cells with nanovectors [21], (v) generation of chimeric antigen receptor (CAR)-T cells [22], and (viii) enhancement of viral episomes when combined with the S/MAR element [23]. Evidently, the S/MAR element is a bona fide modulator of chromatin that confers non-integration as well as mitotic stability to the plasmid derivatives of pEPI-1, in which it is included.

Except for plasmid loss, the low rate of establishment of episomal vectors has been a recurrent issue, holding back the development of gene therapy-oriented episomal vectors. Vector establishment is the process by which the plasmid acquires the state of an autonomous replicon through stochastic epigenetic processes, which allows its faithful replication in eukaryotic cells. The S/MAR-based vectors are stably maintained in the transfected cells in the absence of selection, but their establishment is very inefficient [11]. Establishment sites for S/MAR-based vectors have been mapped by genome-wide profiling of S/MAR replicon contact sites. They are considered nuclear compartments with chromosomal sites involved in extensive gene expression and replication [24]. 

Vector establishment was positively affected by a second chromosomal element, the bona fide mammalian DNA replication initiation region (IR Replicator), which is derived from the 5′ end of the human β-globin gene [25]. It has been shown to have increased potential for replication and transcription, as defined by thermodynamic properties (Stress-Induced Duplex Destabilization Profiles, SIDD) [26]. The addition of the IR replicator to the pEPI-1 vector and the substitution of CMV by the permissive for CD34+ cells SFFV promoter guiding eGFP expression resulted in the construction of the vector pEP-IR [27]. Colony-forming assays of CD34+ cells transfected with eGFP-expressing plasmids showed that cells carrying the PEP-IR vector had a significantly higher number of fluorescent colonies (93%) compared to the colonies derived from the non-IR vector (54%) [27]. Vector pEP-IR proved to be vastly superior compared to the original pEPI-1 vector for gene transfer into CD34+ cells regarding all parameters of episomal gene transfer [27]. The same encouraging results were generated by a third vector namely Zif-VP64-Ep2, based on the S/MAR and the IR chromosomal elements designed to activate the γ-globin gene in CD34+ and other cells [28]. The combination of the S/MAR element with the relatively new IR element in a pPEPI-1 derivative vector displays the best properties for enhanced replication, establishment, non-integration, and mitotic stability as an episomal vector. The data presented herein aim to further establish the functional competence of the S/MAR and IR combinations in CD34+ cells. 

## 2. Materials and Methods

**Figure 1:**
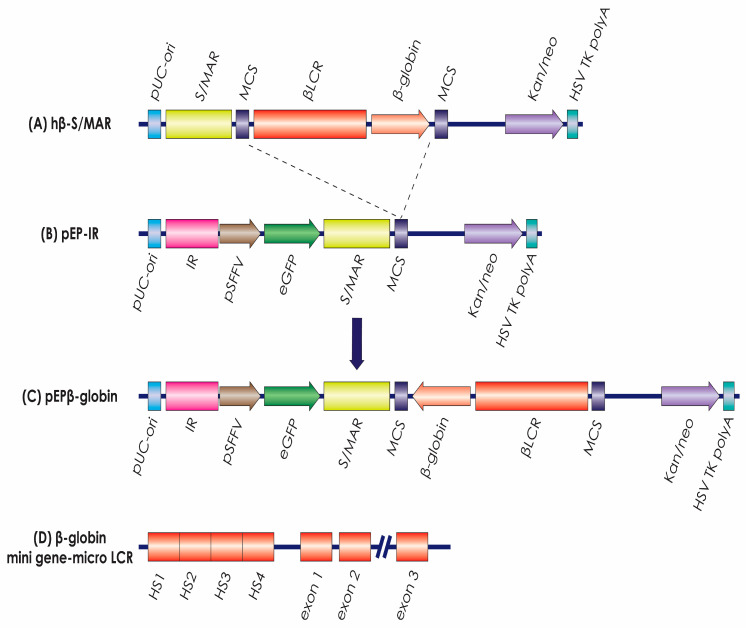
The vectors. Plasmid (**A**) provided the LCR and β-globin genes that were inserted into plasmid (**B**), pEP-IR, to form plasmid (**C**), the experimental vector pEPβ-globin. (**D**) provides the elements of the LCR and of the β-globin gene transferred, (Details in the Section 2).

**Plasmid construction**. Plasmid pEPβ-globin (Figure 1) was constructed by insertion of the ‘micro-LCR mini-HBB gene’ into the linearized plasmid pEP-IR. The plasmid hβ-S/MAR (B) [29] carrying the nearly 8 kb long ‘micro-LCR/mini-HBB gene’ (Figure 1A) was digested with SacII restriction enzyme (Takara Inc., San Jose, CA, USA). Consequently, it was separated by agarose gel electrophoresis and extracted from the gel using the QIAquick Gel Extraction Kit. Plasmid pEP-IR (Figure 1B) was also digested with SacII. The terminal 5′-phosphates were removed by shrimp alkaline phosphatase. The ‘micro-LCR mini-HBB’ gene fragment was inserted into the linear pEP-IR by using a DNA Ligation Kit (Takara) according to the manufacturer’s instructions. The recombinant plasmid transformed competent E. coli cells. The E. coli colonies carrying the plasmid were further expanded in a liquid LB medium with kanamycin. The plasmid was purified using the EndoFree Plasmid Maxi Kit (Qiagen, Venlo, The Netherlands).

**K562 Cell culture and transfection**. Following protocols presented by Stavrou et al. (2017) [27], K562 cells were cultured in DMEM (Gibco, Grand Island, NY, USA) supplemented with 10% FΒS, 2 mM L-glutamine, 50 u/mL penicillin, and 50 mg/mL streptomycin. All reagents were purchased from Invitrogen (Carlsbad, CA, USA). Transfections of K562 cells were carried out by nucleofection using the Amaxa Nucleofector II. Approximately 1 × 10^6^ cells, 1–2 μg plasmid, and 100 μL of the appropriate nucleofection buffer were used in each reaction. Immediately after the procedure, 500 μL of culture medium was added, and the cells were transferred to 6-well plates and incubated for 24 h. The efficiency of the transfections was assessed by flow cytometry as the ratio of GFP-positive (GFP+) cells versus the total amount of live K562 cells. The GFP+ cells were selected by the addition of Geneticin (G418) (Invitrogen) in the medium at a moderate concentration (400 mg/mL). 

**Fluorescence Microscopy and Flow Cytometry**. The eGFP expression of the transfected cells was examined and documented under a fluorescent microscope (Nikon Eclipse TE 2000, Melville, NY, USA). Approximately 3–5 × 10^5^ K562 cells were washed twice with 1ΧPBS and analyzed on a fluorescence-activated cell sorting (FACS) Vantage (BD) flow cytometer, as previously described [28].

**DNA, RNA extraction from K562/eGFP+ cells, and mRNA quantification**. DNA and RNA from K562 transfected cells were extracted with the Allprep DNA/RNA/Protein Minikit (Qiagen, Venlo, The Netherlands), [27,29]. α-, β- and γ-globin mRNA levels were evaluated as follows: RNA (800 ng) extracted from K562 cells transfected with vectors pEPβ-globin or pEP-IR was reverse transcribed to cDNA with Prime-Script TM, a first-strand cDNA Synthesis Kit with Oligo dT primers (Takara Inc., San Jose, CA, USA). For qPCRs, 1/10 of the cDNA was amplified using QuantiFast SYBR Green PCR (Qiagen, Venlo, The Netherlands) in a LightCycler 2.0 (Roche, Vienna, Austria) instrument with the following qPCR protocol: 10 s at 95 °C, followed by 30 s at 60 °C, repeated for a total of 40 cycles. The following primers were used: For the *HBA* gene, Forward: 5′GCACGCTGGCGAGTATGG3′, Reverse: 5′AGGTCGAAGTGCGGGAAGTAG3′. For the *HBB* gene, Forward: 5′GGTCTACCCTTGGACCCAGA3′, Reverse: 5′CAGCAAGAAAGCGAGCTTAGTG3′. For the *HBG* gene, Forward: 5′GACAAGCTGCATGTGGATCCT3′, Reverse: 5′CCGAAATGGATTGCCAAAAC3′. 

For data normalization, the *GAPDH* gene was used as an internal control. The *GAPDH* primers were: Forward: 5′CCATGTTCGTCATGGGTGTGA3′ and Reverse: 5′CATGGACTGTGGTCATGAGT3′. All primers were manufactured by Invitrogen (Carlsbad, CA, USA).

**Plasmid Copy Number estimation**. The plasmid copy number is calculated by performing the procedure according to protocols presented previously [29]. Briefly, total DNA (50–100 ng) was isolated as described above from K562/eGFP+ cells transfected with the vector pEPβ-globin. DNA was amplified using QuantyiFast SYBER Green PCR (Qiagen) in a LightCycler 2.0 (Roche, Vienna, Austria) instrument with the following qPCR protocol: 10 s at 95 °C, followed by 30 s at 60 °C, repeated for a total of 40 cycles. The e*GFP* DNA primers used were: Forward: 5′GACCACTACCAGCAGAACAC3′, Reverse: 5′GAACTCCAGCAGGACCATG3′ (Invitrogen). Copy number estimation was carried out using the Absolute Quantification analysis (Light Cycler Software 4.05, Roche), following the instructions provided by creating Standard Curves with Genomic DNA or Plasmid DNA Templates for qPCR and following the protocol described by Applied Biosystems software accessed in 2015 [30].

In short, the mass of one copy of each plasmid was calculated using the formula m = n.1.096e−21 g/bp (where m = mass, n = genome plasmid size in bp, e−21 = ×10^−21^). Then, from a 200 ng/μL plasmid DNA solution, a series of dilutions were prepared for 10, 10^2^, 10^3^, 10^4^, 10^5^, and 10^6^ copies per reaction. These dilutions were used in the same qPCR protocol with the above-mentioned e*GFP*-specific primers to produce a curve for every copy-number dilution of the plasmid DNA. The plasmid copy number within the DNA fraction prepared from transfected cells was measured against these curves. The plasmid copy number per cell was calculated on the basis that the mass of the diploid human genome is 6.6 pg, as per Stavrou et al. 2017 [27]. 

**DNA fluorescence in situ hybridization (FISH)**. K562 cells were stably transfected with the vector pEPβ-globin at the 14th week of continuous culture. Non-transfected K562 cells were hybridized with the pEP-IR plasmid as a hybridization probe that detects transgenes that are episomally retained or integrated. The probe was labeled with fluorescein-12-2′-deoxy-uridine-5′-triphosphate (Roche, Vienna, Austria) by Nick Translation Reaction (Roche) following the manufacturer’s instructions, as previously reported [27,28]. For internal control, a DLEU (13q14) hybridization probe was used, labeled with fluorescein-12-2′-deoxy-uridine-5′-triphosphate (Roche, Vienna, Austria) and tetramethylrhodamine-5-dUTP (Roche), which detect the endogenous 13q14 locus, giving a double-green and red-signal, respectively, as reported previously [20]. A total of 100 metaphase and interphase spreads were analyzed.

**Plasmid rescue assay**. Genomic DNA from transfected K562 cells was used to transform One Shot™ Stbl3™ *E. coli* Chemically Competent cells (ThermoFisher Scientific, St. Bend, OR, USA), according to the manufacturer’s instructions. Transformed *E. coli* colonies were selected using LB-agar plates containing 30 μg/mL kanamycin (Invitrogen). Plasmid DNA was extracted (QuickLyse© miniprep kit, Qiagen) from randomly selected single resistant colonies and subjected to restriction enzyme analysis with SalI, PciI, and NsiI enzymes. Restriction digests were analyzed by agarose gel electrophoresis at 0.8 g% [27,29].

**Immunofluorescence assay**. Stably transfected K562 cells, at the 14th week of continuous culture, were placed on poly-L-lysine-coated glass coverslips. The cells were fixed for 10 **min** with 4% PFA and permeabilized using 0.3% Triton-X. Then, a blocking buffer containing 10% FBS and 3% BSA was applied for 1 **h**. The cells were incubated overnight with an anti-human β-globin rabbit antibody (Cat # PA5-48233/ThermoFisher Scientific). A second anti-rabbit goat antibody carrying Alexa Fluor 568 (Invitrogen) was added. 

**Isolation of human CD34+ primary cells**. CD34+ cells were obtained from umbilical cord blood derived from the Hellenic Cord Blood Bank of the Biomedical Research Foundation Academy of Athens (BRFAA). The protocol was reviewed and approved by the BRFAA institutional review board (IRB) and the Institutional Ethics Committee [27]. CD34+ cells were isolated from low-density mononuclear cells (MNC) derived from cord blood by density gradient centrifugation (1.077 g/mL Ficoll-Paque, Biochrom, Cambridge, UK) and immunomagnetic selection, using a combination of the Miltenyi CD34 MultiSort kit (Miltenyi Biotec, Hong Kong, China) and the LS Columns (Miltenyi Biotec), in accordance with the manufacturer’s instructions. In all preparations, CD34+ cell purity exceeded 98%, as estimated by flow cytometry [27,28].

**CD34+ Cell culture and transfection**. CD34+ cells were cultured at a concentration of 5–10 × 10^5^ cells/mL in StemSpan SFEM II (StemCell Technologies, Vancouver, BC, Canada) supplemented with the recombinant human (rh) cytokines: 100 ng/mL rh stem cell factor (SCF), 100 ng/mL rh thrombopoietin (TPO), and 100 ng/mL rh Flt-3 ligand (FL) (complete, serum-free medium) (all cytokines purchased from PeproTech). A total of 2–3 × 10^6^ CD34+ cells were transfected in triplicates by nucleofection with either 5 μg of pEP-IR or 8 μg of pEPβ-globin, using the Amaxa Nucleofecto-rII/b (program U008) and Human CD34+ Cells Nucleofector solution, following the company’s instructions.

**CD34+/eGFP+ in Colony-Forming Cell (CFC) Assay**. CD34+/eGFP+ cells were sorted by FACS, and live/dead cells were discriminated by propidium iodide (PI) staining. Selected CD34+ cells expressing eGFP (1 × 10^3^ cells/mL) were plated in methylcellulose medium supplemented with the cytokines MethoCult GF + H4435 (Stem Cell Technologies). After 10–14 days of incubation at 37 °C and 5% CO_2_, cell colonies (BFUEs/CFUEs) were counted by an inverted fluorescence microscope (Leica DM IRES2). Single colonies, after 14 days of incubation, were randomly collected, pooled, and used for DNA and RNA preparation with NucleoSpin RNA XS, the Micro kit for RNA purification, and the NucleoSpin RNA/DNA Buffer Set for parallel RNA and DNA purification (Macherey-Nagel, Düren, Germany).

**mRNA quantification from CD34+/eGFP+ Colonies**. Total RNA (300 ng) from CD34+/eGFP+ colony cells was reverse transcribed to cDNA with the Prime Script ^TM^, first-strand cDNA Synthesis Kit with Oligo dT primers (Takara, Japan). As a negative control, total RNA from non-transfected CD34+ cells was used. For qPCRs, 1/10 of the cDNA was amplified using the KAPA SYBR FAST qPCR Kit (Roche) and the Biorad CFX96 Touch Real-Time PCR Detection System as follows: 3 s at 95 °C, followed by 30 s at 60 °C, repeated for a total of 40 cycles. The following primers were used: *eGFP*_Forward: 5′CAGCCACAACGTCTATATCATG3′, *eGFP* Reverse: 5′CTTGTACAGCTCGTCCATGC3′, *HBB*_Forward: 5′GAAGAGCCAAGGACAGGT3′ and *HBB* Reverse: 5′AGTTCATGTCATAGGAAGGGGGA3′ [27].

## 3. Results

We constructed a new episomal vector, pEPβ-globin, carrying the human physiological β-globin (HBB) gene and the reporter eGFP gene into two separate transcription units. Chromosomal elements S/MAR and IR were also incorporated in the pEPβ-globin episomal vector. Consequently, vector pEPβ-globin was used to transfect cells of haematopoietic origin, namely the established human erythroleukemia K562 cell line and the haematopoietic progenitor CD34+ cells, to investigate the vector’s specific transfection parameters. Additionally, we examined its status inside the nucleus of the recipient cell as well as the capacity of the vector to mediate and maintain long-term retention and expression of the β-globin transgene in the host cell nucleus.

### 3.1. Design and Construction of the Episomal Vector pEPβ-Globin

The new episomal vector, pEPβ-globin, is a derivative of the episomal vector pEP1-eGFP. It was designed to carry the human, physiological β-globin gene as the transgene to be studied and the eGFP gene as a reporter gene. It also carries two human chromosomal elements, the S/MAR and the IR, which are fundamental for the efficient performance of the vector pEPβ-globin (Figure 1). Specifically, the new vector pEPβ-globin contains the following functional and genetic elements:

(A). The backbone of the prototype, episomal vector pEP1-eGFP [10], is exempt from genes encoding viral proteins. The human physiological β-globin gene is represented in the form of ‘LCR-HBB mini-gene.’ This mini-gene contains (i) the β-globin gene and no other globin gene from the β-globin-like gene locus and (ii) the ‘LCR microlocus’ carrying the LCR DNaseI hypersensitive sites HS1, HS2, HS3, and HS4 [29,31].
(B). The transcription cassette ‘SFFV-eGFP-S/MAR-Poly A site’ is driven by the Spleen Focus Forming Virus (SFFV) promoter. This cassette comprises the reporter gene for enhanced Green Fluorescent Protein (eGFP), the human chromosomal element (S/MAR), which normally resides at the 5′ end of the human β-interferon gene, and the SV40 Poly-A site. This way, transcription runs through the S/MAR element, which blocks vector integration into the host cell DNA and ensures long-term nuclear retention of the plasmid [10].
(C). A second human chromosomal element, the replication initiation region (IR element), is derived from the 5′ end of the human β-globin gene, from 5,226,995 to 5,228,615 (HBB ENSG00000244734), or from +75 to the −1545 in relation to the transcription initiation of the β-globin gene. The IR element was used in this study for enhanced plasmid replication and establishment [20,27,28].

The construction of episomal vector pEPβ-globin (Figure 1C) was facilitated by the presence of a single SacII restriction enzyme site in vector hβ-S/MAR (B). The ‘LCR-HBB mini-gene’ was isolated by a SacII digest (Figure 1A) [29] and consequently this DNA fragment was inserted into the single SacII restriction enzyme site within vector pEP-IR (Figure 1B) [27]. The ‘LCR-HBB mini-gene’ is composed of the 6.5 kb LCR microlocus, including DNaseI hypersensitive sites HS1, HS2, HS3, and HS4 (Figure 1D) [31] and the β-globin gene, from −265 bp to +600 bp past the poly(A)-addition site [32], reaching a final size of 1388 kb. The resulting insert size is 7.888 bp, and insertion sites have been verified by DNA sequencing. The ‘LCR-HBB mini-gene’ is carrying an internal deletion in intron 2 of the β-globin gene of approximately ∼600 bp (Figure 1D), but it is preserving the consensus splice sites as a fully functional intron [32]. The total size of the vector pEPβ-globin used in transfections is 16,088 kb. 

### 3.2. Transfection Efficiency and Stable Transformation of K562 Cells 

We performed transfection experiments with pEPβ-globin transferred into human erythroleukemia K562 cells in order to investigate the competence of the novel vector as an episome. The established K562 cell line derives from chronic myeloid leukemia cells. It is mostly triploid or tetraploid, typically including a triplicate of chromosome 11 [33], and cells produce γ-globin instead of β-globin protein even though the cell line is established from an adult patient. Therefore, they are appropriate cells for studying the expression of the transferred β-globin gene. Transfections of K562 cells with the pEPβ-globin vector were performed with the aim of characterising the vector’s potential regarding all critical transfection properties.

Transfections with vector pEPβ-globin (16,088 kb) of freshly grown K562 cells were successfully performed in triplicate experiments by nucleofection (Section 2). Transfection of K562 cells by electroporation is very efficient and common. However, the main parameter affecting the capacity of a vector to transfect K562 cells is the size of the vector [6,9]. The transfection efficiency was estimated 24 to 48 h post-transfection as the percentage of eGFP+ cells over total K562 live cells. An adequate mean value of 24.62 ± 7.7% was assessed and considered successful due to the large size of vector pEPβ-globin.

**Figure 2:**
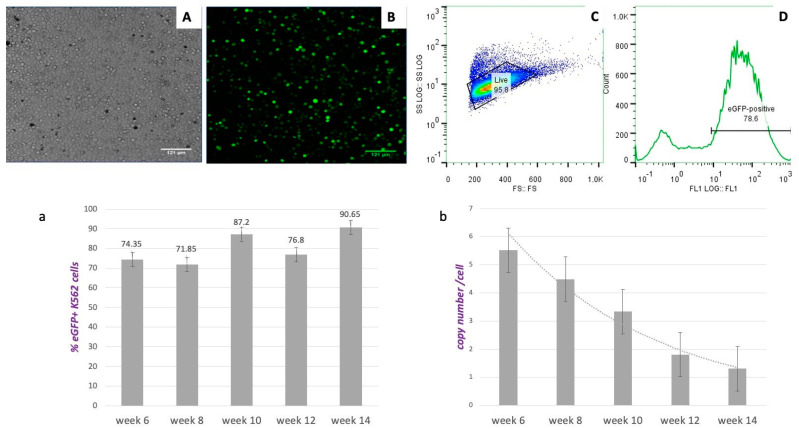
Data from Transfections into K562. Top row: Four weeks of stable culture of transfected cells with vector pEPβ-globin. (**A**) phase-contrast microscopy; (**B**) UV microscopy where only fluorescent cells appear; (**C**) flow cytometry data for the estimation of the percentage of live cells; and (**D**) flow cytometry data for the estimation of the percentage of the fluorescent cells. Bottom row: (**a**) The development of a stable, long-term culture of transfected K562 cells, as determined by the percentage of eGFP+ cells present at each time point; (**b**) the determination and the changes in the copy number of plasmids per cell during long-term culture.

eGFP-expressing (eGFP+) K562 cells carrying vector pEPβ-globin were selected 24 to 48 h post-transfection. Application of G418 for the selection of eGFP+ clones was performed at a medium dose (400 mg/mL) to attain retention of the episomal vector within the host nucleus by offering the advantage of antibiotic resistance. This process lasted for 10 to 14 days. Complete cell death of non-transfected cells was observed in the fourth week of culture from the first application of G418, as has been previously reported [29] and presented in Supplementary Figure S2. A total of 10–14 days post-G418 selection, the transformed K562 cell clones continued growing in G418-free media. Four weeks after G418 pressure relief, the vast majority of transfected cells were growing (Figure 2A), displaying 78.6% of eGFP+ cells (Figure 2B,D). Transformed K562 cell clones during the continuous culture (a total of 14 weeks) were collected for frozen stocks and the isolation of DNA, RNA, and proteins at 2-week intervals. Fluctuation of peaks for eGFP+ transformed K562 cells during the 14 weeks of culture was 71% to 90%, considered statistically insignificant and attributed mainly to sampling errors.

### 3.3. Estimation of Vector pEPβ-Globin Copy Number Per Cell in K562 Transformed Culture

The average number of pEPβ-globin plasmid copies per transformed K562 cell was estimated as stated in Materials and Methods and shown in Figure 2b. During the total culture period of 14 weeks, a slow pace of transient plasmid loss was observed, which could be associated with the large size of the plasmid-vector pEPβ-globin (16,088 kb). Other groups have reported that bigger plasmid vectors maintain a long time in the host nucleus by recruiting histones and escaping nucleases [34]. However, very few of these plasmids are actually established in a stable culture. The number of plasmid copies/per cell was estimated at week 14 of long-term continuous culture to be about 1.4 plasmid copies/cell (Figure 2b). It is noticeable that, while plasmid copies per cell are reduced over time, the transformed K562/eGFP+ cells carrying vector pEPβ-globin are keeping a steady pace of growth and displaying a high percentage of eGFP+ cells (maximum 90.65%) at 14th week during the long-term culture.

### 3.4. Determination of Vector pEPβ-Globin Status into the Nucleus of the Recipient K562 Cells

**Figure 3:**
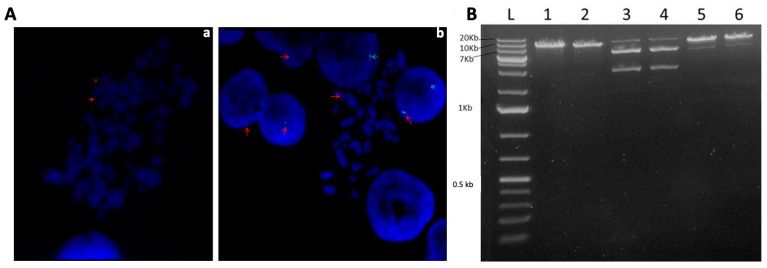
Determination of the episomal status of the vector pEPβ-globin within the transfected K562 cells. (**Aa**,**Ab**) Results of FISH analysis, with red arrows pointing to the green signal of the plasmid and green arrows pointing to dual red and green control signals in metaphases as well as in interphase nuclei. (**B**) presents a plasmid rescue assay with the use of the restriction enzymes SalI, PciI, and NsiI for the digestion of the rescued plasmid DNA lanes 1,3,5 and of the input plasmid DNA lanes 2,4,6 (Detailed explanation in the Section 3).

S/MAR-based vectors are known to be non-integrating, which has been repeatedly shown [10,27,28,35]. However, confirmation of the episomal nature of newly constructed S/MAR vectors is always needed. We performed fluorescent in situ hybridization (FISH) analysis using cells from the long-term G418-free culture transfected with vector pEPβ-globin and non-transfected cells as negative controls at week 14 of culture (the last week before culture termination). FISH analysis was carried out as presented in Section 2, and 30 metaphases and 70 interphase nuclei of transfected cells and an equal number of non-transfected cells were examined. FISH karyotype analyses, either from metaphases or interphase nuclei, showed a specific, single, green signal determining the plasmid DNA existence in the transfected cells in over 90% of the cells examined. In contrast, the signal was detected in different chromosomal positions in K562-transformed cells (Figure 3A). No signal was detected in the non-transfected cells. Internal control green and red dual signals (Section 2) were also observed. In the case of integration events, twin green signals (of integrated vector pEPβ-globin) would be apparent in sister chromatids of the same chromosome after replication during S phase. The FISH analysis did not show such signals and revealed no integration events.

To assess the status of transferred episomes (vector pEPβ-globin) within the K562 cells, we performed a plasmid rescue assay [29]. By this means, genomic DNA from K562 transfected cells from the long-term G418-free culture was isolated in the 14th week and was enriched in plasmid DNA. Hirt extract was used to transform competent E. coli cells in the presence of the proper antibiotic (kanamycin). Only circular, free plasmid molecules existing within the genomic DNA isolated are capable of transforming competent E. coli cells, resulting in colonies on agar plates. Plasmid DNA was extracted from liquid bacterial cultures and digested with the restriction enzymes SalI, NsiI, and PciI. The input plasmid DNA (vector pEPβ-globin) used for transfection of K562 cells was subjected to the same restriction digests as the positive control (Figure 3B). Restriction digestion patterns obtained from the rescued DNA and the input plasmid DNA were identical, providing evidence that the plasmid DNA hosted in transfected K562 cells for 14 weeks of continuous culture (almost 100 new K562 cell generations) remained as a free, circular, plasmid molecule.

### 3.5. Expression of the pEPβ-Globin Transgene in K562 Cells

**Figure 4:**
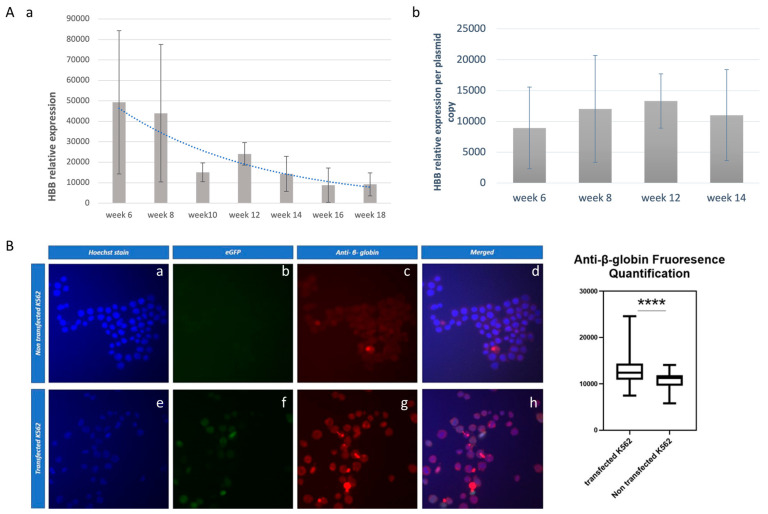
Expression of the β-globin transgene within the K562 cells. (**Aa**) records the β-globin mRNA from week 6 to week 18, estimated as a fold increase in HBB mRNA levels compared to the non-transfected cells, normalized over the expression of the internal gene GAPDH. This vast induction of HBB is due to a low threshold cycle (34.97) of the nearly undetected HBB mRNA levels in the non-transfected cells. (**Ab**) records the mRNA per plasmid copy during the development of stably transfected, long-term cultures. (**B**) depicts the detection of β-globin transgene peptide with the specific β-globin antibody frames f.g.h. and its quantification. **** *p* < 0.0001

The expression of the β-globin transgene during the long-term G418-free K562 transformed cultures, up to week 14, was documented both at the mRNA and protein levels (Figure 4). β-globin mRNA was found to be highly expressed in the 6th week but steadily declined until the 14th week, displaying a ~3,4-fold decrease (Figure 4Aa). The β-globin mRNA decline was parallel to the plasmid copy number decrease obtained at the same time points (Figure 2b). Long-term G418-free K562 transformed cultures were allowed to continue growing for four more weeks, where eGFP+ cell counts at weeks 16 and 18 reached a steady-state level (Figure 4Aa). Noticeably, the β-globin mRNA per vector copy number was steadily maintained (Figure 4Ab).

The full expression of the β-globin mRNA in K562 cells was documented at the protein level using a polyclonal β-globin antibody. This documentation was carried out in stably transfected K562 cells with vector pEPβ-globin at the 14th week of culture (Figure 4B). It resulted in definitively positive results for the β-globin peptide’s successful production within transfected K562 cells. Finally, the abundance of β-globin mRNA against that of α-globin and γ-globin mRNAs was estimated (Table 1). It was shown that the level of γ-globin mRNA far exceeded that of α-globin mRNA—fraction HBG/HBA—in non-transfected and transfected K562 cells, even at the 14th week of the long-term culture. This can be attributed to the nature of K562 cells, which are trisomic for chromosome 11 [33]. HBB mRNA, deriving mostly from the vector transgene, is a 0.13 fraction of the endogenous HBG mRNA at week 8, while the fraction HBB/HBG decreases from week 8 to week 14, as expected from Figure 4Aa. Importantly, the β-globin mRNA corresponds steadily to (roughly) 1/3 of the α-globin mRNA throughout the long-term culture, as shown by the fraction HBB/HBA.

### 3.6. Transfection of Human CD34+ Cells with Vector pEPβ-Globin

Transfection of human haematopoietic progenitor CD34+ cells with the vector pEPβ-globin was carried out by nucleofection to determine the efficacy of transfection and the functional potential of the vector within the hosting CD34+ cells as the main target cell subgroup utilized in gene therapy protocols for haematopoietic disorders.

**Figure 5:**
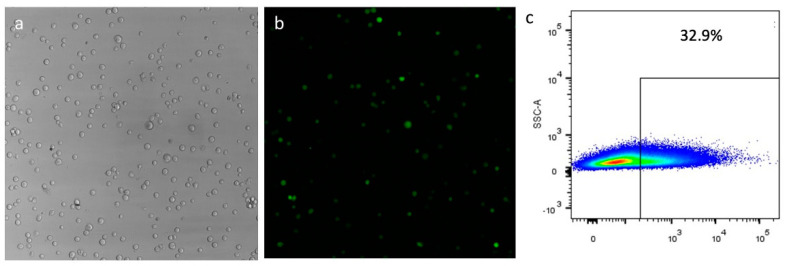
Transfections of CD34+ cells with the plasmid pEPβ-globin 48 h post-transfection; (**a**) shows phase contrast microscopy; (**b**) shows UV microscopy of the same field. (**c**) shows the flow cytometry for the estimation of the transfection efficiency (more data in Supplementary Table S1 and Figure 1).

Human haematopoietic progenitor CD34+ cells were isolated from total mononuclear cells derived from cord blood. They were used in triplicate transfection experiments with vector pEPβ-globin and vector pEP-IR for direct comparisons, as pEP-IR has been previously investigated with clearly positive results [27,28]. Non-transfected CD34+ cells were used as a negative control for β-globin expression. Results from transfections show that vector pEPβ-globin is capable of efficient transfection into CD34+ cells’ nucleus, as previously shown with vector pEP-IR (Figure 5). Mean value efficiencies are 25.64 for vector pEP-IR and 15.57 for vector pEPβ-globin respectively (Supplementary Table S1), estimated at 48 h post-transfection as the number of eGFP-positive (eGFP+) cells over total cells surviving transfection. However, the standard deviation was high for both vectors (Supplementary Table S1). The highest values for transfection efficiencies reached 55.6 for vector pEP-IR and 32.9 for vector pEPβ-globin (Supplementary Table S1; Supplementary Figure S1). The difference in transfection efficiency between the two vectors mirrors the difficulty of large vectors, like pEPβ-globin, to penetrate the cell membrane and the nuclear envelope.

### 3.7. Colony-Forming Cell Assay (CFC) of Transfected CD34+ Cells

**Figure 6:**
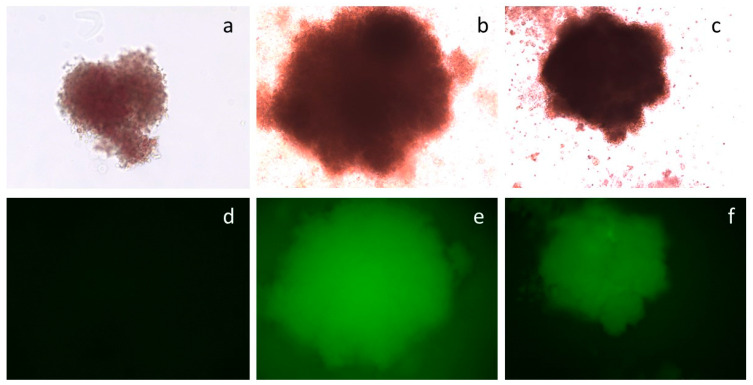
Colonies from the CFC assay of CD34+/eGFP+ cells. Top row, (**a**–**c**): phase contrast microscopy. Bottom row, (**d**–**f**): UV microscopy. Frames (**a**,**d**): non-transfected CD34+ cells. Frames (**b**,**e**): CD34+/eGFP+ cells transfected with plasmid pEP-IR. Frames (**c**,**f**): CD34+/eGFP+ cells transfected with plasmid pEPβ-globin.

A colony-forming cell (CFC) assay is a semi-liquid culture supported with cytokines for the culture of CD34+ haematopoietic progenitor cells. In this culture, each such cell gives rise to a BFU-E/CFUE or CFU-GM differentiated colony over 14 days of culture. Fluorescence-activated cell sorting (FACS) was used to separate CD34+/eGFP+ transfected from non-transfected cells. Both types of cells were placed in CFC assays in triplicates to determine the cells’ capacity for colony formation during the CFC differentiation process towards the erythroid lineage. Results documented that the progeny of the CD34+/eGFP+ cells transfected either with vector pEPβ-globin or with vector pEP-IR were capable of generating transformed fluorescent colonies in CFC assays (Figure 6 and Table 2). The vast majority of the differentiated BFU-E/CFUE/CFU-GM colonies were fluorescent. Noticeably, the percentage of fluorescent colonies with vector pEPβ-globin and those with vector pEP-IR displayed similar mean values: 92.21% and 92.68%, respectively. The remaining colonies in the same culture plates were non-fluorescent; their DNA was tested for eGFP DNA with PCR gene-specific primers, and in both cases of vectors, they were negative for plasmid DNA (Supplmentary Figure S2).

### 3.8. Expression Levels of the pEPβ-Globin Transgene in CFC Assay Colony Cells

It is of considerable significance that vector pEPβ-globin is capable for gene transfer of the β-globin transgene into the CD34+ cells, the main target cells for gene therapy in the haemoglobinopathies. CD34+ cells show basal-level expression of the β-globin gene, which is maintained throughout erythropoiesis and only increases to full expression in differentiated erythroid cells [36]. Differentiated progeny of CFC assays, from non-transfected CD34+ cells to the erythroid lineage, namely the BFU-E/CFUE/CFU-GM cell colonies, express the β-globin gene as a physiological state of their definitive differentiation. Therefore, by performing the CFC assay we intended to estimate the fold change between the β-globin mRNA from CFC colony cells from transfected, fluorescent CD34+/eGFP+ cells and the β-globin mRNA from CFC colony cells from non-transfected, non-fluorescent CD34+ cells.

CFC assays generated BFU-E/CFUE/CFU-GM, fluorescent eGFP+, and non-fluorescent eGFP- colonies from CD34+ cells transfected either with vector pEP-IR or with vector pEPβ-globin; only non-fluorescent colonies were produced from the control non-transfected cells (Table 2).

Expression of the β-globin mRNA (endogenous and exogenous) and the α-globin mRNA (endogenous) was detected in total RNA isolated from transfected CD34+/eGFP+ cells.

**Figure 7:**
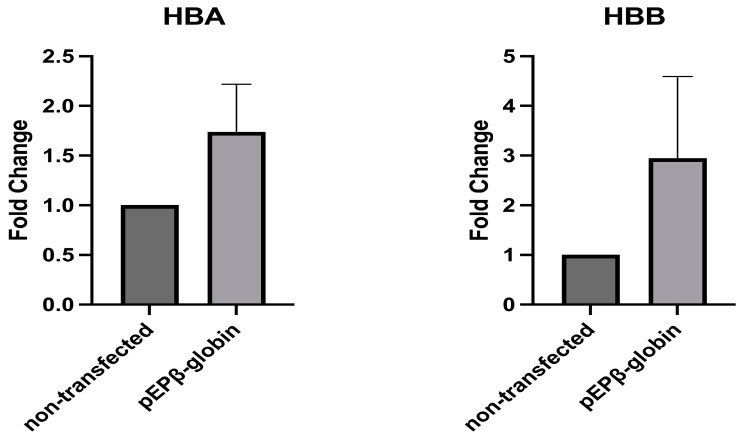
Expression of the α-globin mRNA (HBA) and β-globin mRNA (HBB) within the CFC assay products of CD34+ cells. Statistical analysis of data from triplicate transfections is shown. HBA: Mean = 1.738, SEM = 0.479, Mann–Whitney *t*-test, two-tailed *p* = 0.3143, statistically non-significant. HBB: Mean = 2.949, SEM = 1.644, Mann–Whitney *t*-test, two-tailed *p* = 0.0286, statistically significant (explanations in the Results).

Three separate nucleofections were carried out from three cultures of non-transfected cells (a), CD34+ cells transfected with the vector pEP-IR (b), used as a control regarding the identity and number of colonies produced, and CD34+ cells transfected with the experimental vector pEPβ-globin (c). For each of these transfections, a CFC assay was performed, and differentiated cells were produced. Duplicate samples of pooled fluorescent colonies were taken from each CFC assay. These colonies were derived from CD34+/eGFP+ and non-transfected CD34+ cells. The samples were then used for reverse transcription, followed by qPCR (a total of six PCRs per a, b, and c sets of nucleofections). Important results were obtained, as the estimation of the β-globin mRNA was a 2949-fold increase, and that of the α-globin mRNA was a 1738-fold increase compared to that of the non-transfected CD34+ cells (Figure 7). Both β-globin and α-globin mRNA were elevated in the BFU-E/CFUE/CFU-GM/eGFP+ cell colonies of transfected CD34+. However, only the β-globin mRNA fold change reached statistical significance (Figure 7; Table 2). Non-fluorescent colonies deriving from the same plates of seeded CFC assays CD34+/eGFP+ cells were found to be negative for the vector pEPβ-globin, (Supplement Figure S2).

### 3.9. The Physiological Expression of β-Globin Transgene in CFC Assay Colony Cells

We investigated the possibility that the mRNA values obtained for the β-globin transgene expression are within the range of the physiological expression of this gene (HBB). The physiological range of β-globin gene expression (globin chains) has been reported to be, at birth, between 10% and 20% of the total globin chain synthesis [37,38] (by extrapolation, also in CD34+ cells from human umbilical cord blood isolated upon birth and subjected to CFC assay. We also found that in all cases, the β-globin gene expression in the adult stage reaches 45% of the total globin chain synthesis. This means that from birth to the adult stage, the β-globin gene expression increases by 2.25 to 4.5 fold. Therefore, our estimated 2949-fold increase in β-globin mRNA in the CD34+/eGFP+ CFC colony cells compared to the equivalent CFC assay colony cells from non-transfected CD34+ cells falls well within the adult human physiological range of β-globin expression and is of therapeutic value. This study verifies previous data on the physiological mRNA levels of the same HBB transgene expression from an episomal vector based on the S/MAR element [29].

## 4. Discussion

The first time that the physiological β-globin mini-gene was included and studied in extra-chromosomal DNA—a cosmid of 38 kb containing the native locus control region (LCR), a dominant regulator of the β-globin locus gene expression [39]—in transfections into K562 cells provided clear results for the β-globin transgene expression [9]. In that work, plasmids carrying the individual LCR elements, single or in combination, did not support β-globin expression adequately. The conclusion was that the total LCR was important and necessary for the extra-chromosomal expression of the β-globin gene. This was an impossible condition for the episomal gene transfer of the β-globin gene, if only for the sheer size of the LCR.

The first episomal S/MAR-based vector for the physiological β-globin gene, vector hb-S/MAR (B), accommodating the β-globin mini-gene along with the micro-LCR [29], was used in transfections into K562 cells in which physiological expression of the β-globin gene was documented. Unfortunately, in this work, vector integration events were detected in the third month of continuous G418 culture.

We studied the potential of the episomal vector pEPβ-globin, based on S/MAR and the IR element, for gene delivery of the physiological β-globin gene into the established line of K562 cells and the CD34+ haematopoietic progenitor cells. In the current work, no integration event was found using vector pEPβ-globin in transfection into K652 cells. As the IR element is present only in vector pEPβ-globin and not in vector hb-S/MAR (B), we consider that the ‘’non-integration’’ of vector pEPβ-globin is facilitated by the presence of the IR element. This ensures that every plasmid in every cell, at every replication cycle, is replicating, thus complementing the function of the S/MAR element in maintaining plasmid mitotic stability. We consider that the episomal vector pEPβ-globin is the second-generation vector for the physiological β-globin gene, based on S/MAR and IR elements, after vector hb-S/MAR (B), which was based on the S/MAR element only [29].

Studies with transfections into K562 cells revealed that vector pEPβ-globin carries all the main characteristics of a functional, episomal vector. Specifically, the vector bears important safety features, such as non-integration into the endogenous DNA of the recipient cell and existence as a free, circular, functional plasmid within the transfected cells while, by design, not carrying any protein-coding viral gene. Furthermore, it bears important efficiency features, as it induces stable culture in transfected cells that can acquire significant mitotic stability, supports a long-term culture of stably transfected cells for 18 weeks (Figure 4Aa), and exists at the level of one copy per cell at the stabilized stage. These data are in accordance with previous ones from studies on ‘S/MAR’ and ‘IR’-based vectors [20,27,28].

Vector pEPβ-globin is the first one within the context of S/MAR and IR-based vectors. Along with the eGFP reporter gene transcription cassette (Figure 3), it contains a transcription cassette of a mammalian gene, namely the human β-globin gene, and the micro-LCR as a regulatory element ensuring continuous and stable levels of expression. Our studies on the expression of the β-globin transgene within the transfected K562 cells reveal that vector pEPβ-globin maintains a nearly steady β-globin mRNA per plasmid copy throughout the long-term period of 14 weeks irrespective of changes in its copy number during that period, while both the mRNA and the peptide coded by the β-globin were detected (Figure 4A,B). Therefore, it is documented that vector pEPβ-globin possesses the basic characteristics regarding the safety and efficiency of a valid episomal vector for gene transfer.

The vectors pEP-IR and pEP-βglobin were studied in transfections into human haematopoietic progenitor CD34+ cells, while non-transfected cells were used as controls. The vector pEP-IR has been studied before in CD34+ transfections. Now, it is used as a framework against which the performance of the experimental vector pEPβ-globin is compared. The two functional vectors carry the SFFV promoter to drive eGFP expression (not the original CMV promoter of pEPI-1). This shows that vectors that are used to transfect CD34+ cells need to have a permissive promoter for these cells. Also, it should not be taken for granted that the IR element will have the same effect on other non-haematopoietic progenitor cells, as it is part of the DNA involved in β-globin gene induction and regulation of transcription. Therefore, specific transcription factors may be required for its overall performance. This leads to the suggestion that for a non-CD34+ cell-derived disease, one may need to develop a specific vector with the appropriate combination of S/MAR and the chromosomal element equivalent to IR.

Triplicate experiments’ CFC assays show that the vast majority of CD34/eGFP+ cells transfected either with pEP-IR or with pEPβ-globin produced fluorescent colonies—about 92.4%—(Table 2). Two important points arise from studies on transfected CD34+ cells:

1. Firstly, the results from the CFC assay reveal that the percentage of fluorescent colonies generated by the CD34+/eGFP+ cells carrying two different vectors, namely pEP-IR and pEPβ-globin, are very closely the same. The percentages are 92.68% for pEP-IR and 92.21% for pEPβ-globin, as stated in the Section 3 and can be seen in Table 2. This strongly indicates that the presence of the ‘micro-LCR,’ a chromatin-modifying element (CME), does not offer any particular advantage of performance in CFC assay to vector pEPβ-globin vis-à-vis the vector pEP-IR, which lacks this sequence. A similar situation, regarding the presence or absence of a CME in an episomal vector, has been reported in recent years in the elegant work from the Harbottle laboratory [21], where a nanoSMAR vector that does not include any CME outperforms other episomal vectors with or without a CME. A number of scientific questions spring from these data. For example, is the S/MAR function replacing the LCR function in vector pEPβ-globin? Are the episomal vectors studied here completely free from a chromatin-modifying function in the presence of IR? The answers to such questions are expected to elucidate the role of the IR element in episomal vectors.
2. Secondly, this is the first episomal transfer of the β-globin gene into CD34+ cells, and it is shown that in CFC assay colony cells, the β-globin gene expression, at the mRNA level, is three times higher than that from non-transfected CD34+ cells (Figure 7). This finding verifies previous similar data, obtained for the first time, on the physiological expression of the β-globin gene in a S/MAR-based episomal vector [29].

The CFC culture of 14 days is not, strictly speaking, a long-term culture. Nevertheless, the vector DNA molecules, when they are retained within the CD34+/eGFP+ cells in the nucleus of the recipient cell and they develop their colonies, must undergo a process known as establishment. This process involves acquiring the status of an autonomous replicon, which is defined by epigenetic processes that are largely unknown. This establishment process leads to mitotic stability, ensuring proper segregation of plasmids into daughter cells. These are the two main properties required for the vectors’ long-term retention in the host nucleus [11].

The study of β-globin gene expression in CFC assay colony cells provided excellent results for episomal gene delivery and was highly promising for all CD34+ cell-deriving diseases, primarily haemoglobinopathies. Noticeably, the expression of the β-globin gene reaches closely this level in K562 cell transfections (Figure 4b, week 4).

Furthermore, as episomal plasmid pEPβ-globin carries the ‘micro-LCR,’ the question arises as to whether this element regulates the expression of the neighboring β-globin gene in an episome as it does in a viral vector setting. In the previous paragraph, it was concluded that the ‘micro-LCR’ does not increase the number of fluorescent colonies that appear in a CFC assay compared to that of pEP-IR, but conclusion can be drawn for the β-globin gene expression.

Towards a therapeutic, non-viral, episomal vector for the β-globin gene, the data produced by this study with vector pEPβ-globin provide a solid basis regarding safety and efficiency, but the point of the ‘micro-LCR’ element must be clarified. To this effect, a construct like pEPβ-globin minus the ‘micro-LCR’ can be used in transfections into CD34+ cells and is expected to provide critical data. It is possible that, in the episomal setting, the β-globin gene expression is not affected by the absence of ‘micro LCR.’ Then, the ‘micro LCR’ can be deleted from the plasmid pEPβ-globin, which will reduce the size of the vector by 6.5 kb and augment the resulting plasmid’s transfection efficiency. This is particularly important for the pre-clinical trial, in which the vector has to be transferred into a β-thalassemic mouse model of disease with the aim of correcting the disease. To this effect, a successful transfer into mouse progenitor cells is needed, and the most promising route to this end is currently the use of nanoparticles. If the β-globin gene expression is affected by the absence of ‘micro LCR,’ a new strategy has to be formulated for an episomal vector to be employed.

## 5. Conclusions

It is shown that the second-generation β-globin gene transfer vector, which is a non-viral and episomal vector pEPβ-globin based on S/MAR and IR elements, is non-integrating and exhibits all properties of safety and efficiency. It can be maintained in CD34+/eGFP+ cells and their progeny in CFC assays equally well and no more so than in the established non-viral, episomal vector pEP-IR, rendering the ‘micro LCR’ possibly redundant. Additionally, it is documented that the episomal vector for the physiological β-globin gene, pEPβ-globin, can mediate the physiological level of expression of the β-globin gene within the differentiated progeny of the CD34+/eGFP+ cells at a level of mRNA that is 3-fold higher than the one deriving from the differentiated progeny of the non-transfected CD34+ cells. Therefore, vector pEPβ-globin is an excellent basis for the development of the therapeutic, non-viral, episomal vector for the gene therapy of the β-thalassemia syndromes.

## Figures and Tables

**Table 1:** Estimation of the HBB mRNA transgene production in K562 cells against the mRNA production of the endogenous genes HBA and HBG.

	Non-Transfected Cells	Week 8th	Week 14th
HBG:HBA	4.76 ± 4.47	2.04 ± 3.56	9.03 ± 7.76
HBB:HBG	-	0.13 ± 0.23	0.03 ± 0.03
HBB:HBA	-	0.27 ± 0.23	0.29 ± 0.25

**Table 2:** CFC assays give rise to differentiated colonies from single CD34+/eGFP+ cells. CFC assays were performed for triplicate transfection experiments that included non-transfected CD34+ cells (control), CD34+/eGFP+ cells carrying vector pEP-IR, and CD34+/eGFP+ cells carrying vector pEPβ-globin. CFU-E: Colony-forming unit-erythroid and BFU-E: Burst-forming unit-erythroid, primitive erythroid progenitor cells, CFU-GM: Colony-forming unit-granulocyte monocyte, precursor of monoblasts and myeloblasts. CFU-GEMM: Colony-forming unit-granulocyte/erythrocyte/monocyte/megakaryocyte.

CFC Assay 3 Sets of Nucleofections	CFUE/BFUE	CFU-GM Colonies	CFU-GEMM	Total Colonies	GFP+	GFP+%
a. non-transfected	250	10	30	290	0	0
b. pEP-IR	140	4	8	152	130	85.52
c. pEPβ-globin	40	4	8	52	48	92.30
a. non-transfected	388	52	20	460	0	0
b. pEP-IR	266	10	12	288	270	93.75
c. pEPβ-globin	232	36	20	288	258	89.58
a. non-transfected	113	32	12	157	0	0
b. pEP-IR	150	10	5	165	163	98.78
c. pEPβ-globin	180	25	16	221	210	95.02

## Data Availability

Not applicable.

## Supplementary Materials

The following supporting information can be downloaded at: https://www.mdpi.com/article/10.3390/genes14091774/s1, Figure S1: Flow cytometry of transfection efficiencies. CD34+ cells, non- transfected, transfected with plasmid pEP-IR and transfected with plasmid pEPβ-globin; Figure S2: PCR test for plasmid detection in DNA from CFC colony cells. L. 100bp DNA ladder NEB New England; 1. CFC colony cells transfected with pEP-IR, non-fluorescent; 2. CFC colony cells transfected with pEP-IR, Fluorescent; 3. CFC colony cells transfected with pEPβ-globin, Fluorescent; 4 CFC colony cells transfected with pEPβ-globin, non-Fluorescent; 5. Empty slot; Table S1: Nucleofections to determine transfection efficiencies for transfections into CD34+ cells. Nucleo 1 to 5 are the nucleofections carried out, along with the respective data –columns for non-transfected CD34+ cells, CD34+ cells transfected with plasmid pEP-IR and CD34+ cells transfected with pEPβ-globin. The three last rows provide data from statistical analysis.
