# Supplementary material for: Non-Viral Episomal Vector Mediates Efficient Gene Transfer of the β-Globin Gene into K562 and Human Haematopoietic Progenitor Cells
Source: Genes (Basel). 2023 Sep 8;14(9):1774. doi: 10.3390/genes14091774 (PMC10530965; doi:10.3390/genes14091774)
Supplement: Supplementary file 1 [file genes-14-01774-s001.zip › genes-2438162-supplementary.pdf]

genes

Non-Viral Episomal Vector Mediates Efficient Gene Transfer of the β-Globin Gene into K562 and Human Haematopoietic Progenitor Cells

Vassileios M. Lazaris [1,†], Emmanouil Simantirakis [2,†], Eleana F. Stavrou [1], Meletios Verras [1], Argyro Sgourou [3], Maria K. Keramida [4], George Vassilopoulos [2] and Aglaia Athanassiadou [1,*]

[1] Department of General Biology, Medical School, University of Patras, 26504 Patras, Greece; vlazaris@gmail.com (V.M.L.); stauroue@upatras.gr (E.F.S.); meletver@gmail.com (M.V.)
[2] Centre of Basic Research, Biomedical Research Foundation of the Academy of Athens (BRFAA), 11527 Athens, Greece; esimantirakis@gmail.com (E.S.); gvasilop@bioacademy.gr (G.V.)
[3] Biology Laboratory, School of Science and Technology, Hellenic Open University, 26335 Patras, Greece; sgourou@eap.gr
[4] IVF and Andrology Labs, IVF Unit, General University Hospital of Patras, 26504 Patras, Greece; maria_keram@yahoo.gr
* Correspondence: athanass@med.upatras.gr
† These authors contributed equally to this work.

Supplementary Materials: The following is supporting information.

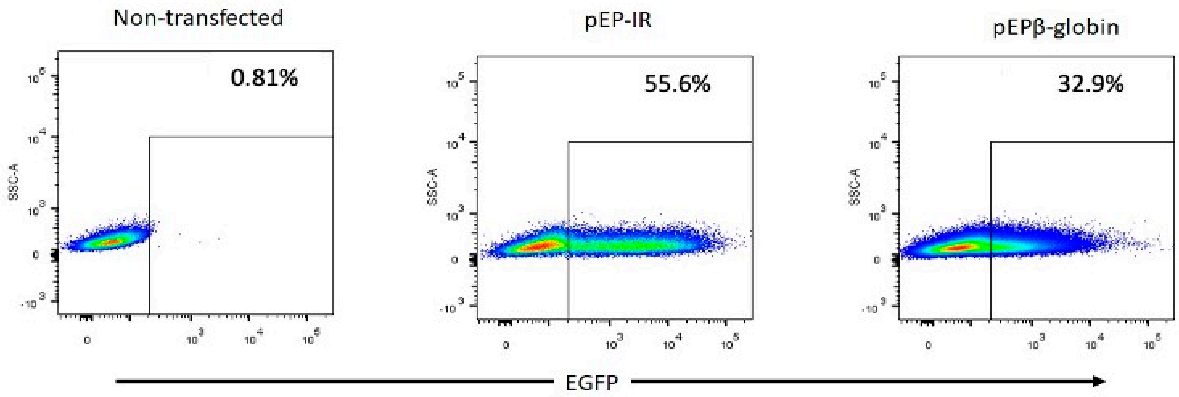

Figure S1. Flow cytometry of transfection efficiencies. CD34+ cells, non-transfected, transfected with plasmid pEP-IR and transfected with plasmid pEPβ-globin.

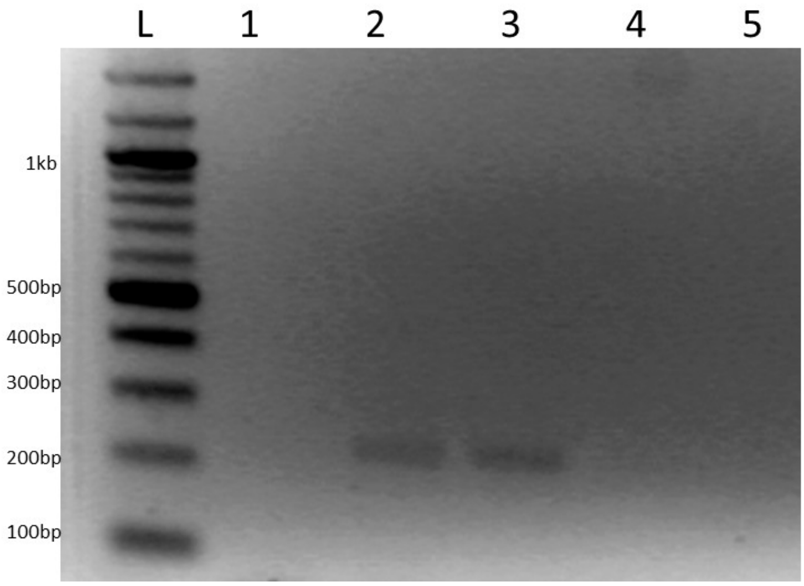

Figure S2. PCR test for plasmid detection in DNA from CFC colony cells. L. 100bp DNA ladder NEB New England; 1. CFC colony cells transfected with pEP-IR, non-fluorescent; 2. CFC colony cells transfected with pEP-IR, Fluorescent; 3. CFC colony cells transfected with pEPβ-globin, Fluorescent; 4 CFC colony cells transfected with pEPβ-globin, non-Fluorescent; 5. Empty slot.

Table S1. Nucleofections to determine transfection efficiencies for transfections into CD34+ cells. Nucleo 1 to 5 are the nucleofections carried out, along with the respective data –columns for non-transfected CD34+ cells, CD34+ cells transfected with plasmid pEP-IR and CD34+ cells transfected with pEPβ-globin. The three last rows provide data from statistical analysis.

Nucleofection	Non- transfected	pEP-IR	pEP-bglobin
nucleo1	0.89	18.3	
nucleo2	1.95	10.2	8.44
nucleo3	0.81	32.6	11.8
nucleo4	1.3	11.5	9.15
nucleo5	0.89	55.6	32.9
average	1.168	25.64	15.5725
Stand-dev	0.47740968	18.9621465	11.6418079
SEM	0.2135041	8.48012972	5.82090396
